# Towards applied swarm robotics: current limitations and enablers

**DOI:** 10.3389/frobt.2025.1607978

**Published:** 2025-06-13

**Authors:** Miquel Kegeleirs, Mauro Birattari

**Affiliations:** IRIDIA, Université libre de Bruxelles, Brussels, Belgium

**Keywords:** swarm robotics, real-world applications, distributed systems, multi-robot systems, design methodology, robot communication

## Abstract

Swarm robotics addresses the design, deployment, and analysis of large groups of robots that collaborate to perform tasks in a decentralized manner. Research in this field has predominantly relied on simulations or small-scale robots with limited sensing, actuation, and computational capabilities. Consequently, despite significant advancements, swarm robotics has yet to see widespread commercial or industrial application. A major barrier to practical deployment is the lack of affordable, modern, and robust platforms suitable for real-world scenarios. Moreover, a narrow definition of what swarm robotics should be has restricted the scope of potential applications. In this paper, we argue that the development of more advanced robotic platforms—incorporating state-of-the-art technologies such as SLAM, computer vision, and reliable communication systems—and the adoption of a broader interpretation of swarm robotics could significantly expand its range of applicability. This would enable robot swarms to tackle a wider variety of real-world tasks and integrate more effectively with existing systems, ultimately paving the way for successful deployment.

## 1 Introduction

Swarm robotics investigates the design, deployment, and evaluation of large groups of robots that collaborate in a decentralized manner to complete their tasks (Dorigo et al., 2014). Research in swarm robotics has rapidly advanced in recent years. One of the main challenges in the field—designing control software for robot swarms—has been thoroughly investigated, with many successful approaches proposed. In particular, (semi-) automatic design (Francesca and Birattari, 2016; Bredeche et al., 2018; Birattari et al., 2019; Birattari et al., 2020) has proven effective in addressing this challenge, with evolutionary robotics (Floreano et al., 2008; Haasdijk et al., 2014; Trianni and López-Ibáñez, 2015; Divband Soorati and Hamann, 2015; Nolfi, 2021) and automatic modular design (Francesca et al., 2014; Spaey et al., 2020; Hasselmann et al., 2021; Mendiburu et al., 2022; Kuckling et al., 2022; Hasselmann et al., 2023; Salman et al., 2024) receiving extensive attention. Researchers have also proposed hybrid solutions that combine evolution and automatic modular design (Ligot et al., 2020; Cambier and Ferrante, 2022; Hasselmann et al., 2023). A promising development is the recent integration of multi-level modeling into the automatic design of control software (Baumann et al., 2022a; Endo et al., 2023). Beyond (semi-) automatic design, various model-based approaches have been proposed, including probabilistic (Correll and Martinoli, 2007; 2011), space-time continuous (Hamann and Wörn, 2008; Hamann, 2010), property-driven (Brambilla et al., 2014), and curiosity-driven models (Kaiser and Hamann, 2022).

Alongside the development of design methods, a better understanding of the reality gap and its effects (Jakobi et al., 1995; Floreano et al., 2008; Francesca and Birattari, 2016) has enhanced their robustness to the sim-to-real transfer. Notably, research has demonstrated that effects akin to those of the reality gap can be replicated in simulation (Ligot and Birattari, 2020), giving rise to the notion of “pseudo-reality.” A pseudo-reality is a simulation model distinct from the one used during the design process. The underlying idea is that control software capable of successfully transferring from the design-phase model to a pseudo-reality is more likely to cross the reality gap than software that cannot (Ligot and Birattari, 2022).

As noted by several authors (Schranz et al., 2020; Dorigo et al., 2021; Dias et al., 2021; Cheraghi et al., 2022; Kuckling, 2023), significant progress has also been made in other areas of the field. Swarms have been developed using underwater (Zahadat and Schmickl, 2016; Connor et al., 2020; Berlinger et al., 2021) and aerial robots (McGuire et al., 2019; Soria et al., 2020; Pavliv et al., 2021; Schilling et al., 2022); heterogeneous swarms have been explored (Dorigo et al., 2013; Zhu et al., 2024); machine learning techniques—such as deep (Hüttenrauch et al., 2017; Hüttenrauch et al., 2019; Yasuda and Ohkura, 2019) and inverse (Gharbi et al., 2023; Szpirer et al., 2024) reinforcement learning—have been integrated into swarm design; advanced capabilities like SLAM (Kegeleirs et al., 2021; Lajoie and Beltrame, 2023) and computer vision (Verlekar and Joshi, 2017; Kegeleirs et al., 2024a; Kegeleirs et al., 2024b) have been tested in swarm contexts; studies on human-swarm interaction have been conducted (Kolling et al., 2016; Podevijn et al., 2016); and swarms of pico-satellites have been investigated (Pinciroli et al., 2008a; Pinciroli et al., 2008b; Fdhila et al., 2012). Moreover, although this paper focuses on macrorobots—i.e., robots at the centimeter to meter scale—swarm robotics holds considerable promise for applications in nanotechnology (Hauert and Bhatia, 2014; Law et al., 2023), albeit with unique challenges of its own.

However, despite these advances, real-world applications of swarm robotics remain extremely limited. To date, no commercial or industrial deployment of robot swarms has been reported. Swarm robotics appears to be at a critical juncture—understanding the reasons for this lack of application and identifying possible enablers could help steer the field toward practical deployment.

## 2 Towards applied swarm robotics

### 2.1 The practical issues

#### 2.1.1 Platform limitations and experimental constraints

A major obstacle to real-world swarm deployment is the lack of modern, affordable, and reliable experimental platforms. Experiments with physical robots remain costly and time-consuming—challenges that scale with swarm size. Researchers also face practical limitations: large spaces are often unavailable or expensive, and acquiring many robots is financially prohibitive. Common platforms used in single- or multi-robot systems are typically too large and costly for swarm use. This has driven the development of swarm-specific platforms that favor low cost and compactness, but at the expense of sensing, actuation, and computational power. The E-Puck (Mondada et al., 2009), Kilobot (Rubenstein et al., 2012), and Crazyflie (Giernacki et al., 2017) are among the most widely used platforms, but they remain limited—plagued by noisy sensors and unreliable actuators. As a result, researchers frequently resort to abstraction (Dorigo et al., 2021), simplifying missions to work around hardware constraints. For instance, two of the most common swarm robotics missions—aggregation and foraging—demonstrate clear limitations.^1^ Aggregation—robots gathering at a single location—is achievable with minimal capabilities but has limited relevance for real-world applications. Foraging—moving objects from one location to another—has greater potential in domains such as logistics, warehouse operations, or search and rescue. Yet, current platform limitations often force researchers to abstract away essential components, such as object manipulation. In both simulation (Wei et al., 2016; Harwell and Gini, 2018; Song et al., 2020; Jimenez Romero et al., 2024) and physical experiments (Rubenstein et al., 2014; Francesca et al., 2015; Hecker and Moses, 2015; Pitonakova et al., 2018; Talamali et al., 2020), robots typically do not carry real objects, undermining the practical credibility of the task. Moreover, while robot swarms typically operate at high densities in lab experiments, envisioned real-world applications often involve sparse swarms, which would call for different control strategies to perform their intended tasks (Tarapore et al., 2020; Kwa et al., 2023b). This results in a disconnect between the long-term ambitions of swarm robotics—space exploration, search and rescue, ocean cleaning—and the highly abstracted, constrained experiments that currently dominate the field. Compounding this issue, platforms like the E-Puck and Kilobot rely on outdated hardware and software architectures. Although efforts have been made to modernize them, such as the Pi-Puck extension for the E-Puck (Allen et al., 2020) or the ROS-ready operating system *DeimOS* (Kegeleirs et al., 2025), they still fall short of overcoming fundamental limitations.

Researchers are often left with two choices: build custom robots or rely exclusively on simulation. Custom-built robots are costly and time-consuming to develop, often tailored to specific research needs—limiting reusability and reproducibility. These efforts are frequently undervalued and rarely supported by thorough documentation. Achieving both capability and compactness is challenging: advanced components demand more space or power, resulting in form factors unsuited to large-scale swarms. Moreover, affordability depends on mass production, which custom robots cannot achieve, while miniaturization requires investments only feasible at industrial scale. Newer platforms such as the S-Drone (Oguz et al., 2022), Mercator (Kegeleirs et al., 2022), and Summit XL (Arregi and Secco, 2023) offer improved capabilities, but none have resolved these trade-offs well enough to gain widespread adoption.

#### 2.1.2 Simulation tools and the deployment gap

Resorting exclusively to simulations is not an ideal solution either. Few simulators are well-suited to swarm robotics. ARGoS3 (Pinciroli et al., 2012) and SwarmLab (Soria et al., 2020), while specifically designed for this purpose, lack the extensive documentation and community support of more general-purpose tools like Gazebo (Koenig and Howard, 2004). Gazebo itself, however, is poorly optimized for large-scale swarm simulations, often struggling to handle more than a few dozen robots efficiently. More critically, the reality gap remains a central concern, especially in the evolutionary approach (Jakobi et al., 1995; Hasselmann et al., 2021). Recent applications of reinforcement learning to robot swarms (Hüttenrauch et al., 2017; Hüttenrauch et al., 2019; Yasuda and Ohkura, 2019) appear to face similar challenges. This is suggested by the scarcity of convincing experiments conducted with real robots in the current literature. More generally, even in single-robot contexts, reinforcement learning methods are known to struggle with sim-to-real transfer due to their sensitivity to modeling inaccuracies and environmental variability (Zhao et al., 2020; Salvato et al., 2021). Consequently, studies conducted solely in simulation provide only limited insight into whether the system will function as expected with real robots. Pseudo-reality (Koos et al., 2013; Ligot and Birattari, 2020) might offer a partial mitigation by exposing controllers to model variations, but it cannot fully guarantee real-world reliability.

Recent studies (Kegeleirs et al., 2024c) have shown that even when a design method succeeds on one physical platform, it may fail to transfer to another. This challenge extends beyond the sim-to-real gap and also affects deployment across physical platforms. We refer to this broader issue as the *deployment gap*: regardless of whether control software is developed in simulation or on a specific robot, its effectiveness is not guaranteed when applied to another platform. Although some methods robust to the reality gap show partial resilience to the deployment gap, further performance degradation still occurs. Consequently, developing control software using overly simplistic robots increases the risk of failure when moving to more capable, field-ready systems.

Resource-sharing infrastructures such as the Robotarium (Wilson et al., 2020) offer partial relief by enabling remote access to real robot swarms. However, these services have notable limitations: users cannot directly interact with the robots or their environment, iterative debugging is more difficult, and demand can restrict timely access. Moreover, the robots—though more modern than Kilobots or E-Pucks—still have limited capabilities, and the system does not easily accommodate complex missions or environmental changes. Scaling up such services would likely require commercial backing, introducing additional costs for users.

#### 2.1.3 Integration challenges: SLAM, vision, and communication

Then, the limitations of current swarm platforms hinder the integration of key robotics technologies such as SLAM, computer vision, and communication. Although its potential was envisioned by early work (Schmickl et al., 2006), only recently has swarm SLAM (Kegeleirs et al., 2021) begun to show practical results (Lajoie and Beltrame, 2023)—and even then, only under constrained, highly structured conditions that are not typical of swarms. Reliable localization remains a significant challenge, especially in the absence of global positioning systems (Quraishi and Martinoli, 2022; Braga et al., 2024). Similarly, while computer vision is ubiquitous in general robotics, it remains underutilized in swarms, largely due to technical limitations. Basic applications have been demonstrated, such as color-based signaling (Nouyan et al., 2009; Chen et al., 2015; Jones et al., 2019; Garzón Ramos and Birattari, 2020) and human-robot interaction based on simple gesture and face recognition (Nagi et al., 2014; Suresh and Martínez, 2019). However, more advanced vision capabilities, like person tracking and re-identification, have only recently been explored in distributed systems (Popovici et al., 2022) and swarms (Kegeleirs et al., 2024a; Kegeleirs et al., 2024b), with modest results so far. Communication also remains underdeveloped (Di Caro et al., 2005; Cianci et al., 2006). Many swarm experiments omit communication altogether, or rely on highly abstracted models such as neighbor detection without actual data exchange. In rare cases, robots share small amounts of numerical data (Ducatelle et al., 2011; Ducatelle et al., 2014; Hasselmann and Birattari, 2020; Garzón Ramos and Birattari, 2020; Talamali et al., 2021; Kuckling et al., 2022). Stigmergy has gained renewed interest (Hunt et al., 2019), but existing implementations often depend on fixed infrastructures that are costly and limited to specific environments (Khaliq et al., 2014; Reina et al., 2021; Na et al., 2021), or materials like wax and alcohol (Russell, 1997; Fujisawa et al., 2014), which pose safety risks due to their flammability and are impractical for most applications. More recently, alternatives based on photo-chromatic pigments have shown potential (Salman et al., 2020; Salman et al., 2024).

Communication between the swarm and external systems—other robots (Dorigo et al., 2013; Kegeleirs et al., 2024b; Zhu et al., 2024) or humans (Nagi et al., 2014; Kolling et al., 2016; Mondada et al., 2016)—is also rare. Again, this is largely due to the lack of suitable communication hardware and protocols. Yet, such capabilities are critical for many envisioned applications.

#### 2.1.4 Regulatory, ethical, and societal barriers

Finally, the deployment of robot swarms remains constrained by ethical and regulatory considerations, particularly regarding their potential ecological and societal impacts (Garzón Ramos and Hauert, 2024; Winfield et al., 2025). This is especially pronounced in the context of aerial drones: although the underlying technology is sufficiently advanced to perform tasks such as aerial surveillance and object detection or recognition, the operation of UAV swarms typically necessitates regulatory waivers and exemptions, which vary significantly across jurisdictions (UK Civil Aviation Authority, 2022; Australian Civil Aviation Safety Authority, 2024; Code of Federal Regulations, 2025). A key factor underlying the reluctance of regulators and operators is the inherent uncertainty surrounding swarm behavior, compounded by a lack of transparency and explainability in their collective decision-making processes (Hussein et al., 2020; Naiseh et al., 2024). Accordingly, it is critical to investigate how swarms are perceived by human users (Carrillo-Zapata et al., 2020) and to develop strategies for fostering (Nam et al., 2019; Lyons et al., 2025) and maintain (Liu et al., 2019) trust, thereby enabling more effective collaboration between humans and robots (Divband Soorati et al., 2022). Public skepticism may also be exacerbated by the increasing use of drones in military contexts, as well as by dystopian portrayals of robots and AI in popular media, both of which may hinder acceptance of swarm technologies in everyday settings.

### 2.2 The conceptual issues

#### 2.2.1 Rigid interpretations of swarm principles

At a conceptual level, swarm robotics often clings to foundational conventions, treating core principles as fixed rules and defaulting to standard design choices without critical reflection. The canonical definition emphasizes fault tolerance, flexibility, and scalability—emerging from redundancy, self-organization, and locality of sensing and communication (Dorigo et al., 2014). These principles have driven progress but can become limiting when treated as strict requirements. Due to technical and economic constraints, achieving them in practice is often difficult, prompting researchers to simplify experiments just to preserve the “swarm” designation. Moreover, while these features offer clear advantages, they are often seen in industry as impractical compared to centralized, high-performance systems. This rigidity discourages hybrid approaches that might be more viable in real-world settings. A swarm can—and arguably should—leverage centralized components when useful, without losing its distributed character.

#### 2.2.2 Verification and assumptions about swarm properties

Equally important, fault tolerance, flexibility, and scalability are often assumed rather than formally or empirically verified. Formal verification remains a major challenge. Early approaches based on temporal logic (Rouff et al., 2004; Winfield et al., 2005; Dixon et al., 2012; Gjondrekaj et al., 2012) are highly sensitive to the state explosion problem, limiting their scalability. Later methods—such as probabilistic model checking (Konur et al., 2012), statistical model checking (Massink et al., 2013), and property-driven design (Brambilla et al., 2014)—enabled more scalable analyses but often lacked consistent implementation of system models that support practical simulation and testing. More recent techniques come with trade-offs: some require extensive expert knowledge (Coppola et al., 2019), others focus solely on software-level verification (Merlo et al., 2022), and some reintroduce scalability issues (Leofante et al., 2019). Consequently, researchers often fall back on qualitative demonstrations—for example, showing stable performance across different swarm sizes or different environments, or resilience to robot failures. Recent findings indicate that scalability (Kuckling et al., 2024) and possibly other key properties (Hunt et al., 2019) may have practical limitations—even in systems specifically designed to exhibit them. While these properties are definitely an asset of robot swarms, making unexamined assumptions about them without rigorous validation risks misleading future research.

#### 2.2.3 Isolation vs integration with external systems

Rigid thinking in swarm robotics also affects how swarms are composed and interact with other systems. Most studies focus on homogeneous swarms operating in isolation. Even heterogeneous swarms—where different types of robots collaborate (Ducatelle et al., 2011; Dorigo et al., 2013)—are typically treated as self-contained entities (Kwa et al., 2020; Wang et al., 2021; van Diggelen et al., 2024), broadening capabilities but not addressing isolation from external actors such as humans, other robots, or machines. Although some exceptions exist (Zhu et al., 2024), it remains rare for swarms to operate alongside—let alone in support of—other systems. Yet, one of their key strengths is distributed environmental sensing: swarm robots can rapidly gather and update mission-specific data through peer-to-peer sharing (Jones et al., 2020). This *swarm perception* is often studied in the context of collective behavior (Brambilla et al., 2013; Trianni and Campo, 2015) and decision-making (Valentini et al., 2016b; Valentini et al., 2016a; Strobel et al., 2018; Zakir et al., 2022), but its potential to assist external agents remains underexplored (Naghsh et al., 2008; Kegeleirs et al., 2024b). Acknowledging that swarms need not be self-contained could unlock a wide range of new applications.

#### 2.2.4 Overlooked aspects: navigation, heterogeneity, and data security

Several critical topics remain underexplored. Navigation, for instance, is often treated as an implementation detail, despite its central role in robotic behavior and its influence on experimental outcomes. Random walk is the default strategy in many studies, yet this term covers a range of behaviors—Brownian motion (Feynman et al., 2011), correlated random walk (Renshaw and Henderson, 1981), Levy walk (Zaburdaev et al., 2015), and Levy taxis (Pasternak et al., 2009)—each with different performance characteristics depending on the platform and context. For instance, a configuration optimized for Kilobots (Dimidov et al., 2016) performs poorly on E-Pucks (Kegeleirs et al., 2019). More advanced strategies such as flocking (Hauert et al., 2011; Toshiyuki et al., 2016; Baumann et al., 2022b; Brandstätter et al., 2024) and connected locomotion (Mamei et al., 2004; O’Grady et al., 2009; Slavkov et al., 2018; Carrillo-Zapata et al., 2019) are promising alternatives and deserve further attention.

Finally, secure data storage and sharing (Hunt and Hauert, 2020) remains overlooked in robot swarms. Their decentralized nature provides inherent advantages: sensitive data is fragmented, stored locally, and often shared as processed outputs rather than raw streams—all of which reduce vulnerability to unauthorized access. Still, swarms are susceptible to attacks, including infiltration by byzantine robots (Strobel et al., 2023) or physical capture of units to access onboard data. Practical deployments rely on centralized infrastructure, introducing additional risks when interfacing with external systems. Blockchain-based solutions have been proposed (Dorigo et al., 2024), but robust, field-tested security mechanisms for swarms remain an open challenge.

### 2.3 Key enablers for real-world deployment

#### 2.3.1 Bridging the deployment gap

From a practical standpoint, swarm robotics research must align more closely with real-world conditions. First, providing evidence that a robot swarm can bridge the deployment gap should become standard practice. It remains uncertain whether findings obtained on current research platforms are transferable to the advanced robots required for real-world applications. In particular, it is unclear whether artificial evolution could effectively generate behaviors for more capable, sophisticated swarms. Hence, simulation experiments should be systematically validated by real-robot experiments or, at a minimum, in pseudo-reality (Ligot and Birattari, 2020; Ligot and Birattari, 2022). In addition, hardware-in-the-loop approaches can also yield valuable insights and strengthen the connection between simulation and reality (Zhang et al., 2020; Khaliq et al., 2021; Jiang and Patil, 2022). Automatic modular design approaches like AutoMoDe (Birattari et al., 2019; 2021) have shown promise in narrowing the deployment gap (Francesca et al., 2014; Kegeleirs et al., 2024c). Another promising strategy is to use a smaller, less powerful platform as a proxy for a more advanced one (Kegeleirs et al., 2024c). If control software can transfer between the two, the smaller platform can be used for large-scale testing—albeit with limited capabilities.

#### 2.3.2 Modernizing swarm platforms

Second, standard research platforms in swarm robotics should evolve toward modern, more capable robotic systems. Hardware for sensing, actuation, and computation has become increasingly compact and affordable. Additionally, adopting standard frameworks—such as ROS—or developing alternatives (Baumann and Martinoli, 2021) would enhance robot capabilities, encourage benchmarking, and improve reusability of research outputs. The long-standing image of swarm robots as simplistic, near-useless individuals—once a powerful metaphor for emergent intelligence—is now becoming a liability. Even without competing with industrial systems, designing research-dedicated robots with similar capabilities would support emerging technologies like swarm SLAM and enable the integration of computer vision. More capable platforms would also allow researchers to design more complex missions that demand richer and more relevant behaviors. In particular, robot-to-robot communication should be more prominent in experiments to fully leverage collective intelligence. Collaboration with industry could help align platform design with real-world needs while contributing valuable expertise and technical resources.

Moreover, the concept of robot swarms can extend to systems beyond the traditional focus on mobile robots—whether terrestrial, aerial, or aquatic. Stationary systems—including intelligent structures, embedded objects, or even non-autonomous robots like wearables—can also operate as swarms. For example, a swarm of smart solar panels could use self-organization and self-assembly to maximize energy production. Recent studies also envision swarms of intelligent objects in artistic and architectural applications (Alhafnawi et al., 2021), or to enhance human-swarm interaction in activities such as brainstorming and opinion gathering (Alhafnawi et al., 2022).

#### 2.3.3 Breaking swarm stereotypes

Conceptually, the idealized definition and role of robot swarms should be re-examined to better leverage their unique strengths. Even systems that only partially conform to traditional swarm constraints can offer significant value. A semi-autonomous swarm guided by a leader—be it a centralized system or another robot—can still exhibit self-organization, redundancy, and local interactions at the agent level. Such swarms may be ideally suited for environments like warehouses or monitoring systems. Recent research on *ad hoc* hierarchical structures emerging through self-organization offer another promising direction, closer to swarm definitions (Mathews et al., 2017; Zhang et al., 2023).

The expected level of flexibility in swarms may also warrant reconsideration. While adaptability is a clear strength—especially in unknown environments—many real-world applications involve (semi-)structured settings where adapting the environment to suit the swarm may be more practical. Indeed, adapting the environment to accommodate robotic systems is already common in domains such as social and assistive robotics (Šabanović, 2010; Kyrarini et al., 2021; Tsunoda and Premachandra, 2021; Kodate, 2023; Yoshikawa, 2024). In light of these shifting assumptions and hybrid designs, researchers should be more deliberate in defining the properties they expect from swarm systems and should more frequently employ formal verification methods. To support this, the development of standardized metrics and evaluation frameworks is particularly important (Ferreira Cruz et al., 2021; Kwa et al., 2023a; Milner et al., 2023).

#### 2.3.4 Rethinking swarm’s role

Expanding beyond traditional swarm structures—through heterogeneity or support for external systems—could greatly broaden the field’s applications. In particular, leveraging swarm perception to collect and relay data for other systems is highly promising. A swarm can act as a distributed sensor network, continuously collecting and updating mission-specific environmental data. Although such data is usually used internally to refine collective behavior, it can also be viewed as a shared, dynamic environmental database. Providing this information to external systems—for instance, through communication with a separate agent—could supply critical data for completing other tasks. For instance, a robot swarm may not be ideal as a standalone search-and-rescue solution, but could still play a vital role by supporting human rescuers.

Human-swarm interaction already partially explored this concept (Kolling et al., 2016; Hussein and Abbass, 2018), enabling operators to use swarm-generated information to improve safety and efficiency. For example, a rescuer could locate victims based on swarm data, or a speleologist might rely on swarm-generated maps to plan an exploration.

Finally, swarm SLAM holds strong potential for supporting external systems. While swarms typically excel at creating coarse, abstract maps—less useful within the swarm—they are ideal for scouting missions where the swarm’s objective is to quickly relay basic mapping information to another system. Such maps can provide valuable navigational support for other robots or situational awareness for human operators, underscoring swarm SLAM’s role in exploration and reconnaissance.

Ultimately, realistic applications will require swarm robotics to integrate modern technologies and rethink some of its core assumptions. Combined with SLAM, tracking individuals across large spaces is one particularly promising use case. Multi-target, multi-camera tracking (MTMCT) and person re-identification (Re-ID) remain challenging, especially in uncontrolled environments (Amosa et al., 2023; Tang et al., 2017; Ristani and Tomasi, 2018; Gaikwad and Karmakar, 2021; Ye et al., 2022). Robot swarms offer a unique advantage: they can reposition themselves to overcome occlusions and capture richer visual data, in particular in unknown environments where strategies dependent on fixed sensor placements or path planning are impractical (Robin and Lacroix, 2016). They can also share data in real time to maintain robust identification and localization, enabling support for other robots’ navigation. For example, a hospital delivery robot could use swarm-generated data to locate the requesting doctor. In surveillance or crowd monitoring, swarms could cover blind spots left by fixed infrastructure. Crucially, swarm robots can do more than detect—they can act. Unlike passive systems, swarms can initiate local responses—either mitigating an issue until human intervention arrives or resolving it autonomously.

## 3 Conclusion

In this paper, we have reviewed the key challenges that currently limit the adoption of swarm robotics in real-world applications (see Table 1). In particular, progress is hindered by the lack of affordable, modern research platforms and by a rigid adherence to conventional definitions of swarm robotics. As a result, many swarm experiments remain overly simplistic and offer limited guarantees of reproducibility on real or more sophisticated robotic systems. There is also a tendency to overestimate swarm properties without sufficient empirical validation. We argue that developing reliable, modern platforms—potentially through industry collaboration—would empower researchers to perform more realistic and impactful experiments, accelerating progress in navigation, vision, and communication. We further contend that rethinking the conceptual foundations of swarm robotics could open up novel application domains. In particular, loosening strict adherence to traditional swarm principles could significantly broaden the scope of the field. For example, developing semi-autonomous swarms or swarms designed to support external systems offers promising pathways toward real-world deployment.

**TABLE 1:** Practical and conceptual barriers in swarm robotics, and their corresponding enablers.

Category	Barrier	Key enabler
Practical	Outdated platforms with limited sensing, actuation, and computation	Develop modern research platforms with enhanced sensors and computing capabilities
	Simulator limitations and deployment gap	Apply pseudo-reality testing, hardware-in-the-loop validation, and platform generalization techniques
	Poor integration of SLAM, vision, and communication	Embed advanced SLAM, vision, and communication stacks in new standard platforms
	Regulatory, ethical, and trust-related concerns	Promote transparency, human-swarm trust, and early engagement with regulators
Conceptual	Rigid adherence to canonical swarm properties	Rethink the paradigm: allow hybrid or leader-guided designs while preserving decentralization
	Unverified assumptions about swarm properties	Introduce formal validation, empirical testing, and standardized performance metrics
	Isolationist mindset (self-contained swarms only)	Reposition swarms as task enablers or data providers within broader multi-agent systems
	Overlooked aspects (e.g., navigation strategies, heterogeneity, security)	Prioritize these topics to enable richer, more realistic applications and robust deployments

## Data Availability

The original contributions presented in the study are included in the article/supplementary material, further inquiries can be directed to the corresponding authors.

## Footnotes

1 These missions are extensively cited in the literature (Brambilla et al., 2013; Bayındır, 2016; Nedjah and Silva Junior, 2019; Schranz et al., 2020; Calderón-Arce et al., 2022), with foraging receiving special attention (Lu et al., 2020).
